# Physician-complementing artificial intelligence in haematology: ushering in a new era

**DOI:** 10.1038/s41375-026-02864-y

**Published:** 2026-01-29

**Authors:** Junren Chen, Robert Peter Gale

**Affiliations:** 1 State Key Laboratory of Experimental Hematology, National Clinical Research Center for Blood Diseases, Haihe Laboratory of Cell Ecosystem, Institute of Hematology & Blood Diseases Hospital, Chinese Academy of Medical Sciences & Peking Union Medical College, Tianjin, China; 2 Tianjin Institutes of Health Science, Tianjin, China; 3 Centre for Haematology, Department of Immunology and Inflammation, Imperial College of Science, Technology and Medicine, London, UK

**Keywords:** Biotechnology, Medical research

Use of artificial intelligence (AI) in haematology is advancing rapidly. Initially, AI was used for pattern recognition, like analysing blood and bone marrow slide images and radiographs. Now, use has expanded to analysing multi-dimensional clinical data to assist in diagnosis, estimating prognosis, guiding therapy decision-making, and prescribing drugs [1–3].

There is considerable debate whether AI should be *physician-substituting* or *physician-complementing* [4, 5]. *Substituting* and *complementing* are economic concepts best illustrated by examples (Fig. 1). Assume, on a scale of 100, the performance level of skilled physicians is 70 and that of less-skilled physicians, 50. An AI technology increasing their performance to 80 and 70 shrinks the performance gap to 10 and is termed *physician-substituting* because it disproportionally makes up for a shortage of skills in less-skilled physicians. In contrast, an AI technology which increases the level of skilled and less-skilled physicians to 90 and 60 widens their performance gap to 30 and is termed *physician-complementing* because it disproportionately increases the advantage of skilled physicians.

**Fig. 1:**
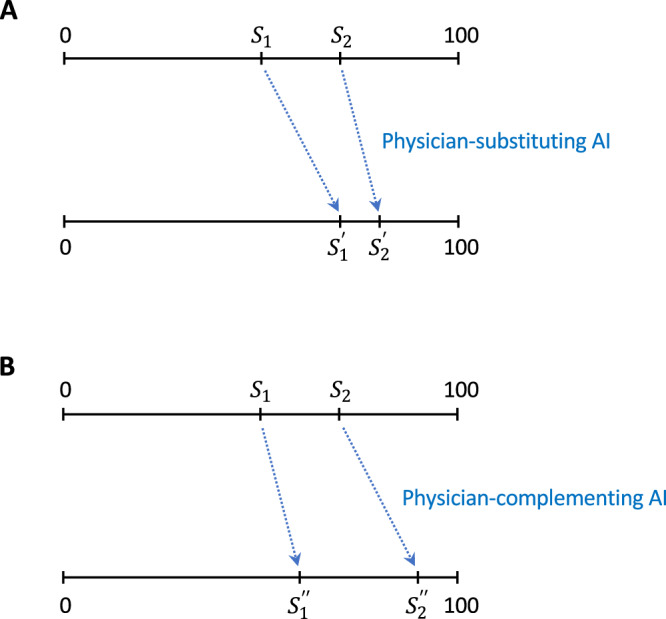
Physician-substituting versus -complementing artificial intelligence. **A** ${S}_{1}$ and ${S}_{2}$ are performance levels of less-skilled and skilled physicians, respectively, without artificial intelligence assistance. A technology that raises their performance to ${S}_{1}^{{\prime} }$ and ${S}_{2}^{{\prime} }$ and shrinks the gap between them is *physician-substituting*. **B** On the other hand, a technology that raises their levels to ${S}_{1}^{{\prime} {\prime} }$ and ${S}_{2}^{{\prime} {\prime} }$, thereby amplifying their performance gap, is *physician-complementing*. Our definition of *substituting* and *complementing* follows ref. [29].

Physician-substituting and -complementing AI technologies are not mutually exclusive. Without resource constraints, we can use physician-substituting AI for less-skilled haematologists and physician-complementing AI for skilled haematologists. However, if we must choose one AI technology, the decision will depend on whether it is more important to improve the basic level of healthcare quality (i.e., developing physician-substituting AI for less-skilled haematologists) or its maximum height (i.e., developing physician-complementing AI for skilled haematologists). Almost everyone, including physicians, tends to rate themselves as above average, but daily experience is also fraught with a sense of inadequacy and insecurity [6]. This raises the difficult question: What is the goal of AI in haematology? Is it to raise the performance of less-skilled haematologists to near that of skilled haematologists, or something else? Different people have different answers, but *a rising tide lifts all boats* [7]. Physician-substituting AI has the potential to elevate healthcare standards in resource-constrained areas. However, it is equally important to push the performance frontier forward, and for this we argue that physician-complementing AI can play an important role.

Physician-complementing AI empowers skilled physicians to do more and makes them even better at what they do. This does not imply that a physician-complementing AI technology necessarily engages in direct patient care. Consider ambient documentation technology, which focuses on other tasks [8, 9]. Haematologists, regardless of their level of empathy, spend considerable time during a patient encounter on non-patient-facing tasks like documenting the visit in the electronic medical record (EMR) and (re)-ordering laboratory tests and drugs. AI scribes can assume these tasks, allowing haematologists with more empathetic skills to increase their interaction with patients.

We anticipate that many physician-complementing AI technologies will directly contribute to therapy decision-making and drug prescribing. One area where even highly-skilled haematologists may need help is *precision medicine*, wherein every person’s therapy should consider their unique features, such as genetic variant topography and co-morbidities. As more and more covariates are considered, the *curse of dimensionality* quickly arises. Even the most intelligent and skilled person cannot consciously process many variables simultaneously because conscious thoughts have a limited bandwidth [10, 11]. The low capacity for conscious deliberation forces decision-makers to focus on a subset of variables with imperfect weighting, distorted by anchoring and recall bias heuristics. In our RAND-Delphi study of breast cancer therapy, haematologists/oncologists claimed they needed data on 15 covariates before opining whether an autotransplant is appropriate therapy for a specific patient. However, when we analysed their recommendations using recursive partitioning, we found they used only 3 or 4 covariates to guide their decision-making [12].

With AI assistance, haematologists will be empowered to consider a much greater number of covariates compared with those they can currently process. Skilled haematologists would be able to process all available clinical and laboratory data when deciding whether to prescribe a drug, because an AI agent is embedded in the EMR to assist them. One example is a recent prospective trial of using autonomous AI to monitor a very large number (>140) of dynamic clinical covariates and assist transplant experts in making complex decisions on pre-emptive drug intervention to prevent grade III–IV acute graft-versus-host disease in people receiving a human leucocyte antigen-haplotype-matched haematopoietic cell transplant [3]. Most physicians and patients agreed to let AI prescribe the drug, even though they were free to modify or decline the AI model’s  recommended prescriptions.

Another potential use case for physician-complementing AI might be deciding whether to recommend intensive induction chemotherapy (cytarabine/daunorubicin) or less intensive therapy (azacitidine/venetoclax) to someone >65 years old with acute myeloid leukaemia, especially if they are scheduled to subsequently receive a haematopoietic cell transplant [13]. In addition, if they achieve a histological complete remission, would they benefit from more chemotherapy pretransplant? [14] Should results of measurable residual disease- and/or genetic variant topography-testing influence this decision? [15–19] There are many covariates the haematologist should consider in recommending a therapy strategy [20]. AI might assist the haematologist and improve his/her therapy recommendation, an assumption testable in a randomised controlled trial (RCT).

Another example is deciding whether to prescribe an immune therapy drug for someone with Hodgkin lymphoma and a pre-existing autoimmune disorder, wherein it is important to balance benefits and risks but there are few data to inform decision-making [21, 22]. Physician-complementing AI might help haematologists by summarising evidence from diverse data sources and EMRs of similar people with Hodgkin lymphoma receiving immune therapy, thereby providing *structure* in considering huge numbers of covariates.

In addition to clinical literature data (expert consensus statements, clinical practice guidelines, results of RCTs, etc.), skilled haematologists consider many more covariates, including their experience with similar patients and therapies (recall heuristic), concern for *quality-of-life* (QoL), doctor-patient interactions, and confidence in their ability to decide on a therapy plan [23]. However, efficacy endpoints in haematology trials are often progression-free survival (PFS) and/or survival. Expert consensus statements and clinical practice guidelines usually assume these endpoints are what people value most, shifting the burden of evidence review from patients and physicians to experts [24]. Nevertheless, people may rank other outcome attributes above survival [25]. For example, some may prefer a therapy with less time spent in healthcare facilities compared with another therapy associated with longer survival but more time in healthcare facilities [26]. Yet others may prioritise avoiding pain and/or avoiding adverse events over longer PFS or survival. Haematologists should adjust therapy decisions based on these considerations [27]. However, currently there is no established best practice for this task [28].

*Patient preference-sensitive decision-making* can be broken down into 2 steps: (1) identifying a patient’s preferences; and (2) prioritising therapy options based on their preferences. For the 1st step, AI can assist the haematologist and patient to take part in a structured conversation to *discover* the patient’s weighting of outcome attributes, including survival, treatment response, risk of recurrence, therapy-related adverse events, QoL, modes of care delivery, burden on caregiver(s), and stress related to time and cost. *Discovered* patient preferences can be cross-checked with previous EMRs. For instance, AI perusal of EMRs might reveal frequent prior hospital and/or clinic visits related to therapy-related adverse events, perhaps corroborating a patient’s stated low tolerance of intensive therapy. Subsequently, in the 2nd step, another AI module ranks available therapy options based on the patient’s preferences. However, currently, expert consensus statements and clinical practice guidelines prioritise PFS and/or survival over other outcome attributes and do not support preference-sensitive decision-making because they are not structured in a way that metadata of therapy options beyond PFS and survival are easily queryable. Responsibility of future task forces for drafting clinical practice guidelines will include systematic review and compilation of benefits and harms of therapy options, rather than prescribing one *optimised* patient care pathway assuming a universal preference for PFS or survival.

There are caveats to our forecast of the future of AI in haematology. 1st, we write from the perspective of high-resource, tertiary medical centres with sophisticated EMRs with ready interoperability with AI systems. Our forecast might not operate at all hospitals and clinics. 2nd, presently, most medical AI studies are not done in prospective settings, and performance is often measured on highly-curated datasets using metrics not directly connected to patient well-being. Prospective testing of model performance using credible benchmarks is needed to persuade sceptics. 3rd, throughout our discussion, we assume AI is a co-pilot partnering with haematologists rather than a fully autonomous arbitrator of medical decision-making. Whatever recommendations AI makes have to be ratified by physicians in accordance with current regulations in most jurisdictions. The more complex a decision-making setting, the more necessary the requirement for physicians to have the competence to evaluate recommendations generated by AI. A natural corollary to this is that the most sophisticated AI models are necessarily physician-complementing rather than -substituting because they can be safely used only by skilled physicians. And 4th, for rare diseases and/or new therapies, there might be insufficient data to support preference-sensitive decision-making even if we can reliably measure patients’ outcome preferences. Under these circumstances, decision-making needs to rely on skilled physicians’ judgement.

There is consensus that AI will transform haematology. Exactly how and when are being debated. With modern cooking techniques (e.g., *sous vide*, flash freezing, and ultrasonic homogenising), master chefs create new, previously unthinkable dishes. We suggest that physician-complementing AI will allow haematologists to take patient care to new heights.

## References

[1] Nazha A, Elemento O, Ahuja S, Lam B, Miles M, Shouval R, et al. Artificial intelligence in hematology. Blood. 2025;146:2283–92. 40845137 10.1182/blood.2025029876

[2] Liu X, Cao Y, Guo Y, Gong X, Feng Y, Wang Y, et al. Dynamic forecasting of severe acute graft-versus-host disease after transplantation. Nat Comput Sci. 2022;2:153–9. 38177449 10.1038/s43588-022-00213-4 PMC10766514

[3] Chen J, Cao Y, Feng Y, Qi S, Yang D, Hu Y, et al. Autonomous artificial intelligence prescribing a drug to prevent severe acute graft-versus-host disease in HLA-haploidentical transplants. Nat Commun. 2025;16:8391. 40998766 10.1038/s41467-025-62926-0 PMC12462483

[4] Hooker RS. Physician substitution is undergoing evolution and change. JAAPA. 2021;34:11. 34524162 10.1097/01.JAA.0000791504.22100.29

[5] Sezgin E. Artificial intelligence in healthcare: complementing, not replacing, doctors and healthcare providers. Digit Health. 2023;9:20552076231186520. 37426593 10.1177/20552076231186520 PMC10328041

[6] Davidai S, Deri S. The second pugilist’s plight: Why people believe they are above average but are not especially happy about it. J Exp Psychol Gen. 2019;148:570–87. 30802129 10.1037/xge0000580

[7] The Laymen in New York. Boston Evening Transcript. 22 January 1910.

[8] Tierney AA, Gayre G, Hoberman B, Mattern B, Ballesca M, Kipnis P, et al. Ambient artificial intelligence scribes to alleviate the burden of clinical documentation. NEJM Catal Innov Care Deliv. 2024;5:CAT-23.

[9] Olson KD, Meeker D, Troup M, Barker TD, Nguyen VH, Manders JB, et al. Use of ambient AI scribes to reduce administrative burden and professional burnout. JAMA Netw Open. 2025;8:e2534976. 41037268 10.1001/jamanetworkopen.2025.34976 PMC12492056

[10] Halford GS, Baker R, McCredden JE, Bain JD. How many variables can humans process?. Psychol Sci. 2005;16:70–6. 15660854 10.1111/j.0956-7976.2005.00782.x

[11] Dijksterhuis A, Bos MW, Nordgren LF, van Baaren RB. On making the right choice: the deliberation-without-attention effect. Science. 2006;311:1005–7. 16484496 10.1126/science.1121629

[12] Gale RP, Park RE, Dubois R, Bitran JD, Buzdar A, Hortobagyi G, et al. Delphi-panel analysis of appropriateness of high-dose chemotherapy and blood cell or bone marrow autotransplants in women with breast cancer. Clin Transplant. 2000;14:32–41. 10693633 10.1034/j.1399-0012.2000.140107.x

[13] Rodriguez-Arboli E, Gale RP. Does everyone with newly-diagnosed, untreated acute myeloid leukaemia need remission-induction chemotherapy before advancing to a transplant?. Leukemia. 2025;39:2858–61. 41083680 10.1038/s41375-025-02784-3

[14] Copelan EA, Avalos BR, Gale RP. Pretransplant conditioning for hematopoietic cell transplants: the past is prologue. Transplant Cell Ther. 2025;31:857–62. 40714366 10.1016/j.jtct.2025.07.016

[15] Othus M, Gale RP, Hourigan CS, Walter RB. Statistics and measurable residual disease (MRD) testing: uses and abuses in hematopoietic cell transplantation. Bone Marrow Transplant. 2020;55:843–50. 31666655 10.1038/s41409-019-0729-4 PMC7462748

[16] Venditti A, Gale RP, Buccisano F, Ossenkoppele G. Should persons with acute myeloid leukemia (AML) in 1st histological complete remission who are measurable residual disease (MRD) test positive receive an allotransplant?. Leukemia. 2020;34:963–5. 32132654 10.1038/s41375-020-0780-6

[17] Gale RP, Phillips GL, Lazarus HM. A modest proposal to the transplant publik to prevent harm to people with acute myeloid leukaemia in 1st complete remission cured by chemotherapy. Leukemia. 2024;38:1663–6. 38459165 10.1038/s41375-024-02214-w PMC11286528

[18] Chen J, Gale RP, Hu Y, Yan W, Wang T, Zhang W. Measurable residual disease (MRD)-testing in haematological and solid cancers. Leukemia. 2024;38:1202–12. 38637690 10.1038/s41375-024-02252-4 PMC11147778

[19] Wei AH, Iland HJ, DiNardo CD, Reynolds J. MRD intervention in AML: a new therapeutic horizon. Blood. 2026;147:13–23. 10.1182/blood.2025029010 41105915

[20] Fenwarth L, Thomas X, de Botton S, Duployez N, Bourhis JH, Lesieur A, et al. A personalized approach to guide allogeneic stem cell transplantation in younger adults with acute myeloid leukemia. Blood. 2021;137:524–32. 32871585 10.1182/blood.2020005524

[21] Yue Q, Deng X, Xu Y. Limitations in quantitative harm-benefit assessment in immuno-oncology. JAMA Oncol. 2025;11:1549. 41066097 10.1001/jamaoncol.2025.3786

[22] Heyward J, Segal JB. Limitations in quantitative harm-benefit assessment in immuno-oncology-reply. JAMA Oncol. 2025;11:1550–1. 41066098 10.1001/jamaoncol.2025.3792

[23] Nilsson MS, Pilhammar E. Professional approaches in clinical judgements among senior and junior doctors: implications for medical education. BMC Med Educ. 2009;9:25. 19460139 10.1186/1472-6920-9-25 PMC2693513

[24] Krahn M, Naglie G. The next step in guideline development: incorporating patient preferences. JAMA. 2008;300:436–8. 18647988 10.1001/jama.300.4.436

[25] Shen Q, Gale RP, Chen J. Rethinking end points in modern oncology trials-beyond the P value. JAMA Oncol. 2025;11:1553. 41165689 10.1001/jamaoncol.2025.4340

[26] Gupta A, Eisenhauer EA, Booth CM. The time toxicity of cancer treatment. J Clin Oncol. 2022;40:1611–5. 35235366 10.1200/JCO.21.02810

[27] Wiercioch W, Morgano GP, Piggott T, Nieuwlaat R, Neumann I, Sousa-Pinto B, et al. GRADE Guidance 42: using thresholds for judgments on health benefits and harms in decision making (GRADE Guidance 42). Ann Intern Med. 2025;178:1644–52. 41052449 10.7326/ANNALS-24-02013

[28] Wickramasekera N, Shackley P, Rowen D. Embedding a choice experiment in an online decision aid or tool: scoping review. J Med Internet Res. 2025;27:e59209. 40117570 10.2196/59209 PMC11971581

[29] Becker GS. A Treatise on the Family. Enlarged ed. Harvard University Press, Cambridge, 1993.

